# Structural consequence of the most frequently recurring cancer-associated substitution in DNA polymerase ε

**DOI:** 10.1038/s41467-018-08114-9

**Published:** 2019-01-22

**Authors:** Vimal Parkash, Yashraj Kulkarni, Josy ter Beek, Polina V. Shcherbakova, Shina Caroline Lynn Kamerlin, Erik Johansson

**Affiliations:** 1 Department of Medical Biochemistry and Biophysics, Umeå University, Umeå, SE-90187 Sweden; 2 Department of Chemistry - BMC, Uppsala University, Box 576, Uppsala, S-751 23 Sweden; 3 Eppley Institute for Research in Cancer and Allied Diseases, Fred & Pamela Buffett Cancer Center, University of Nebraska Medical Center, Omaha, NE 68198 USA

## Abstract

The most frequently recurring cancer-associated DNA polymerase ε (Pol ε) mutation is a P286R substitution in the exonuclease domain. While originally proposed to increase genome instability by disrupting exonucleolytic proofreading, the P286R variant was later found to be significantly more pathogenic than Pol ε proofreading deficiency *per se*. The mechanisms underlying its stronger impact remained unclear. Here we report the crystal structure of the yeast orthologue, Pol ε−P301R, complexed with DNA and an incoming dNTP. Structural changes in the protein are confined to the exonuclease domain, with R301 pointing towards the exonuclease site. Molecular dynamics simulations suggest that R301 interferes with DNA binding to the exonuclease site, an outcome not observed with the exonuclease-inactive Pol ε−D290A,E292A variant lacking the catalytic residues. These results reveal a distinct mechanism of exonuclease inactivation by the P301R substitution and a likely basis for its dramatically higher mutagenic and tumorigenic effects.

## Abstract (web summary)

Mutations in the human *POLE* gene are associated with tumours with high mutational loads. Here the authors provide a structural rationale for the mutagenic activity of the cancer-associated DNA polymerase ε P286R variant.

## Introduction

Accurate duplication of DNA is a prerequisite to prevent mutations in the genome, which may result in tumor development and other diseases. At the eukaryotic replication fork, the family B replicative DNA polymerases epsilon (Pol ε) and delta (Pol δ) synthesize the leading and lagging strand, respectively^1^. Both are high-fidelity polymerases, and their remarkable replication accuracy is due to the combined action of accurate selection of nucleotides by its highly selective polymerase site (Pol site) and removal of incorrect nucleotides by the exonuclease domain^1,2^. In addition, mismatch repair removes any incorrect nucleotides that have escaped the proofreading activity and further reduces DNA duplication errors^2,3^.

The exonuclease domains of family B polymerases have several highly-conserved motifs (named Exo-motifs^4^), which include the catalytic residues (D290, E292, D383 and D477 in yeast Pol ε). The catalytic residues serve as ligands for two metal ions^5^, and substituting both D290 and E292 with alanine abolishes the proofreading function of the polymerase, which in turn increases the in vitro mutation rate by ~100-fold^6^. Studies in yeast, mice and human cells have demonstrated that inactivation of the exonuclease activity in Pol ε leads to an increased spontaneous mutation rate^7–9^. The increased spontaneous mutation rate in mice with both alleles of *Pole* encoding for a proofreading-deficient Pol ε also led to the development of tumors^8^.

An analysis of human cancer exomes revealed an increased prevalence of mutations in the *POLE* gene encoding Pol ε in a subset of colorectal and endometrial tumors with a very high mutation load^10–13^. The most prevalent change was the replacement of P286 by arginine, and several other substitutions within the Pol ε exonuclease domain have also been seen recurrently. The location of the affected amino acid residues in or around the exonuclease active site led to the idea that the pathogenic effects of *POLE* mutations stem from their adverse effects on Pol ε proofreading activity^14–16^. This idea was initially supported by the demonstration of severely reduced exonuclease activity of several cancer-associated Pol ε variants, including P286R^17^. However, studies in the yeast model showed that the *pol2-P301R* allele (corresponding to human *POLE-P286R*) has a 50-fold higher mutator effect than the *pol2-4* allele encoding exonuclease-inactive Pol ε (D290A,E292A)^18^. Similar higher mutator effects of the P286R variant are seen in mouse embryonic fibroblasts, and mice with the germline *Pole*^*P286R*^ mutation are dramatically more cancer-prone than *Pole* exonuclease-deficient mice^19^. Taken together, these observations suggest that P286R substitution must have distinct consequences than simple inactivation of the exonuclease, and these other consequences must be important for its role as a potent cancer driver. The structural and mechanistic basis for the astoundingly strong in vivo effects of Pol ε-P286R remains unexplored.

**Fig. 1:**
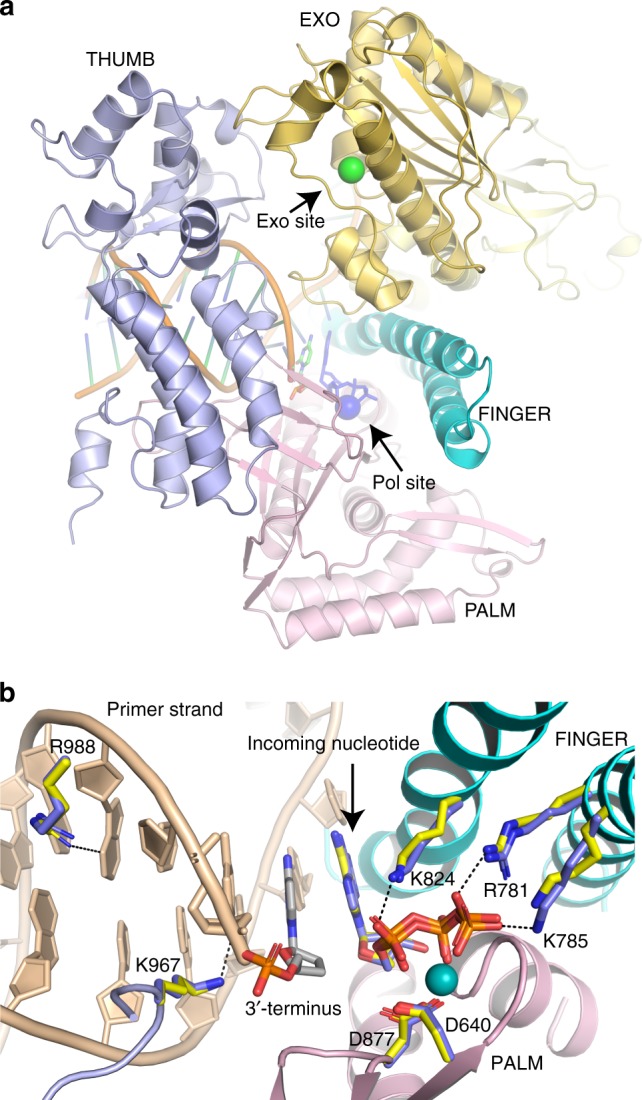
Structure of Pol2_CORE_ P301R. **a** Overview of the Pol ε structure with the defined domains. The P-domain is not visible from this view as it is located on the other side of the structure. The position of the polymerase active site and exonuclease active site is indicated with a blue and green sphere, respectively. **b** Superimposition of the Pol2_CORE_ P301R structure (yellow sticks) on the previously published Pol ε structure (4m8o[https://www.rcsb.org/structure/4m8o]^26^) (blue sticks). The incoming nucleotide at the polymerase site is stabilized by the metal in the B-site, K824, R781 and K785 from the finger domain. R988 and K967 stabilize the 3´-end of the DNA in the polymerase active site^28^. D877 and D640 are the catalytic residues

At present, many structures of family B DNA polymerases with proofreading capacity have been determined, and their overall structures are well conserved, consisting of a thumb, palm, fingers and the exonuclease domain. Among the family B polymerases, RB69 gp43 is the best characterized with structures available of the polymerase in different modes, such as the apo-enzyme with no DNA bound (PDB IDs: 1waf [https://www.rcsb.org/structure/1waf]^20^ and 1ih7[https://www.rcsb.org/structure/1ih7]^21^), as well as RB69 gp43 in complex with DNA in the polymerase mode (PDB ID: 1ig9[https://www.rcsb.org/structure/1ig9]^21^), and with DNA in the editing mode (PDB IDs: 1clq[https://www.rcsb.org/structure/1clq]^22^ and 2p5o[https://www.rcsb.org/structure/2p5o]^23^). These structures provided insight into the mechanism of DNA synthesis and proofreading by this class of DNA polymerases and have served as a paradigm for understanding the eukaryotic replicases. In RB69 gp43, the polymerase and the exonuclease sites are located about 35–40 Å apart in separate domains, necessitating a major structural change in the enzyme for switching from polymerization to proofreading (distance shown for Pol ε in Fig. 1a). In the process, 3–4 base pairs are separated to allow the single-stranded DNA to bind to the exonuclease site^22,24,25^. There are two published crystal structures of the catalytic part of the Pol2 subunit (Pol2_CORE_) of *S. cerevisiae* Pol ε in a ternary complex with DNA in the polymerase mode (PDB IDs: 4m8o[https://www.rcsb.org/structure/4m8o]^26^ and 4ptf[https://www.rcsb.org/structure/4ptf]^27^), which reveal similar arrangement of the polymerase and exonuclease domains. How this arrangement and the enzyme’s active sites are affected by the cancer-associated amino acid substitutions has not yet been determined.

Here we report the crystal structure of the catalytic core of *S. cerevisiae* Pol ε with a P301R substitution, orthologous to P286R in human Pol ε. The new structure, in combination with molecular dynamics simulations, reveals major differences between Pol ε-P301R and the catalytically inactive Pol ε-D290A,E292A in the mechanism of exonuclease inactivation, and helps explain the vastly stronger mutagenic and tumorigenic effects of the cancer variant.

## Results

### The overall structure of yeast Pol ε P301R

To investigate the structural and functional impact of the P301R mutation on *S. cerevisiae* Pol ε, the catalytic core of the Pol2 subunit of Pol ε (Pol2_CORE_) with the P301R substitution was purified. The Pol2_CORE_-P301R was stable during the purification, gave a similar yield as the wild-type Pol2_CORE_, and crystallized readily in the presence of DNA and an incoming nucleotide. The structure of Pol2_CORE_-P301R was superimposed on previously solved structures of Pol2_CORE_ — PDB 4m8o[https://www.rcsb.org/structure/4m8o]^26^ and 4ptf[https://www.rcsb.org/structure/4ptf]^27^—with a root mean square deviation (r.m.s.d.) of less than 0.5 Å. There were no rearrangements of the N-terminal, exonuclease, finger, palm, thumb or P-domains (Supplementary Figure 1), nor were there any alterations in the interactions with DNA and the incoming nucleotide in the ternary complex of Pol2_CORE_-P301R (Fig. 1). The polymerase active site is identical to that in 4m8o[https://www.rcsb.org/structure/4m8o]^26^ and 4ptf[https://www.rcsb.org/structure/4ptf]^27^, and the position of K967 and R988, which stabilize the 3´-end in the polymerase site^28^, also remains unchanged (Fig. 1b). All considered, this suggests that the capacity of Pol2_CORE_-P301R to synthesize DNA should not be compromised.

### Impact of the P301R substitution on the exonuclease domain

**Fig. 2:**
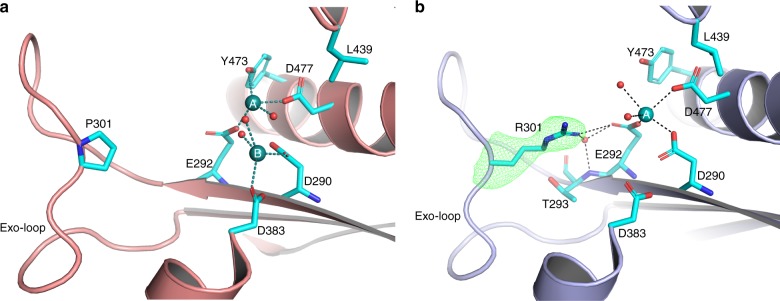
The wild-type and P301R exonuclease active site of Pol ε. **a** The crystal structure of Pol2_CORE_-M644G showing the active site of the wild-type exonuclease domain (shown in salmon) with two metals (in dark cyan) and the Exo-loop with P301. Residues mentioned in the main text are shown in cyan. **b** The structure of the exonuclease domain of Pol2_CORE_-P301R (shown in light blue) showing the active site and the Exo-loop. The polder omit map calculated for R301 at 5σ is shown as a green mesh. Residues mentioned in the main text are shown in cyan. The metal Ca^2+^, located in the A-site, is shown in dark cyan

The catalytic site of the exonuclease domain is supposed to harbor two metal ions, which are essential for the exonuclease activity^5^. The published Pol ε wild-type exonuclease domain structure, solved by Jain et al.^27^, only possesses one metal ion, located at the B-site. Within another project we solved a Pol2_CORE_ structure of a polymerase active site mutant, Pol2_CORE_ M644G, which has an unaffected wild-type exonuclease domain. This structure of the wild-type exonuclease domain, solved at 2.5 Å resolution, contained two Ca^2+^ ions located at the A- and B-site of the exonuclease site (Fig. 2a). We used this structure to assess the impact of P301R substitution on the exonuclease domain and will refer to it as wild type for the purpose of exonuclease domain comparison. In contrast to the wild-type, the P301R structure harbors only a Ca^2+^ ion in the A-site, coordinated by water molecules and three catalytic residues D290, E292 and D477 (Fig. 2b). The B-site was not occupied by a metal in the P301R structure (Fig. 2b).

The overall crystal structure of the exonuclease domain of Pol2_CORE_-P301R is very similar to the wild-type structure (Supplementary Figure 1). The P301R substitution is located in a loop, called the Exo-loop, but does not affect the overall conformation of the Exo-loop. In the Pol2_CORE_-P301R structure, the R301 points toward the catalytic site, forming an ion pair with E292. The R301-E292 ion pair interaction is buttressed by a water molecule that is stabilized by the backbone amide group of T293 (Fig. 2b). Another crystal structure of Pol2_CORE_-P301R in a different space group—PDB 6i8a[https://www.rcsb.org/structure/6i8a], Table 1—also showed a very similar ion pair interaction between R301 and E292.

**Table 1:** Data Collection and refinement statistics (molecular replacement)

	Pol2_CORE_-M644G PDB ID 6fwk	Pol2_CORE_-P301R PDB ID 6g0a	Pol2_CORE_-P301R PDB ID 6i8a
Data collection			
Spacegroup	P2	C2	P2
Cell parameter			
*a,b,c* (Å)	154.0, 70.3, 158.9	159.9, 67.25, 151.6	154.5, 70.3, 159.3
*α*, *β*, *γ* (°)	90, 112.8, 90	90, 111.5, 90	90, 112.8, 90
Resolution range (Å)	50–2.5(2.57–2.5)	78–2.62(2.71–2.62)	50–2.65(2.81–2.65)
Completeness (%)^a^	99.2(94.1)	98.7(98.2)	98.7(94.5)
*I/σ(I)* ^*a*^	8.76(0.63)	5.9(1.3)	10.23(0.89)
*R*_meas_ (%)^*a*^	10.8(196)	15.6(105.4)	8.9(142.5)
Refinement			
Resolution (Å)	2.5	2.62	2.65
No. of reflections	107685	44625	90534
*R*_work_*/R*_free_*(%)*	21.8/26.3	22.4/26.5	22.9 (27.9)
No. of atoms			
Protein	17295	8625	16937
DNA	1056	528	1056
dATP	60	30	60
Metal	7	3	6
Water	11	15	6
B factors			
Protein	73.7	54.9	78.7
DNA	64.5	39.82	68.0
dATP	50.7	30.65	53.6
Metal	81.5	59.90	83.6
Water	69.4	42.5	72.4
R.m.s deviations			
Bond length (Å)	0.005	0.006	0.006
Bond angle (°)	0.736	0.830	0.795

^a^Values in parentheses are for the outer shell. X-ray data were collected from single crystal

### A model to assess the impact of P301R on DNA binding

**Fig. 3:**
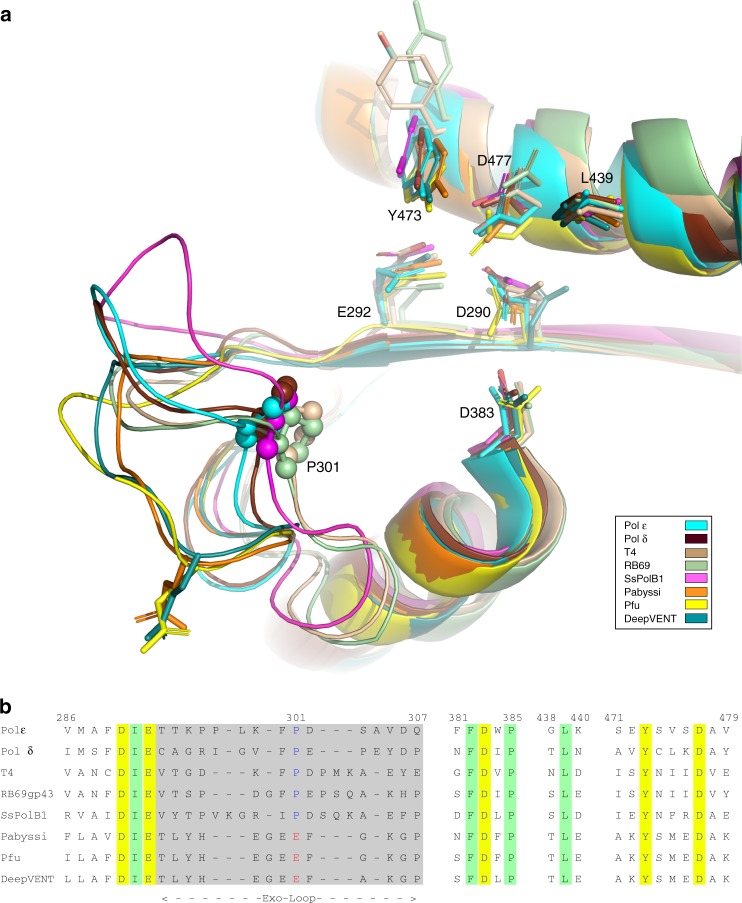
Structural superimposition and sequence alignment of exonuclease domains from family B polymerases. **a** Superimposition of the structures of RB69 gp43 (1clq [https://www.rcsb.org/structure/1clq]^22^, pale green), T4 DNA polymerase (1noy [https://www.rcsb.org/structure/1noy]^38^, wheat), *S. cerevisiae* Pol2_CORE_ (6fwk [https://www.rcsb.org/structure/6fwk] from this study, cyan), *S. cerevisae* Pol δ (3iay [https://www.rcsb.org/structure/3iay]^36^, chocolate), Deep VENT (5h12 [https://www.rcsb.org/structure/5h12]^35^, dark cyan), *S. solfataricus* PolB1 (1s5j [https://www.rcsb.org/structure/1s5j]^34^, magenta), *P. furiosus* DNA polymerase (2jgu [https://www.rcsb.org/structure/2jgu]^31^, yellow) and *P. abyssi* DNA polymerase (4flw [https://www.rcsb.org/structure/4flw]^37^, orange). **b** Structure based sequence alignment. Residues involved in the catalytic activity are shown in yellow background. The length of the Exo-loop is marked with arrows. P301 is in blue font, and the corresponding glutamic acid is in red. Other conserved residues are in pale green background

In the absence of a structure of Pol ε with DNA in the exonuclease site, we must rely on molecular dynamics (MD) simulations, in which the single-stranded DNA (ssDNA) is artificially modeled into the exonuclease site of the wild-type Pol *ε* structure, for further structural and dynamical insights. To select a starting model for the ssDNA in the exonuclease domain of Pol2, we performed a BLAST^29^ search against the PDB database^30^ with the *S. cerevisiae* Pol2 exonuclease domain (PDB 4m8o [https://www.rcsb.org/structure/4m8o]^26^) as a reference, to compare with other solved DNA polymerase structures. We found the exonuclease domains of archaeal DNA polymerases: *P. furiosus* polymerase (PDB 2jgu [https://www.rcsb.org/structure/2jgu]^31^, 3a2f [https://www.rcsb.org/structure/3a2f]^32^, 4ahc [https://www.rcsb.org/structure/4ahc]^33^), *S. solfataricus* PolB1 (PDB 1s5j [https://www.rcsb.org/structure/1s5j]^34^) and Deep vent DNA polymerase (PDB 5h12[https://www.rcsb.org/structure/5h12]^35^), to have the highest sequence identity (about 27%) with the exonuclease domain of Pol2. In addition, we expanded the structural analysis of the exonuclease domain to other family B polymerases with poor sequence conservation (sequence identity less than 15%), such as Pol δ (PDB 3iay [https://www.rcsb.org/structure/3iay]^36^), RB69 gp43 (1clq [https://www.rcsb.org/structure/1clq]^22^), *P. abyssi* B family polymerase (PDB 4flw [https://www.rcsb.org/structure/4flw]^37^) and T4 DNA polymerase (PDB 1noy [https://www.rcsb.org/structure/1noy]^38^). Despite the very low sequence identity, the fold of the exonuclease domains of these DNA polymerases is very similar (Fig. 3a).

The active site residues D290, E292, D383 and D477 (yeast Pol ε sequence numbering) are strictly conserved in all family B DNA polymerases (Fig. 3). In addition, Y473 and L439 residues close to the active site are conserved (Fig. 3b). However, the Exo-loop is poorly conserved (Fig. 3b), even though it influences the shape of the cavity where ssDNA binds to the exonuclease site by stacking the terminal nucleotide of ssDNA. Both the length and choice of amino acids in the loop varies among different polymerases, but they fall into two classes. (1) Pol ε, Pol δ, RB69 gp43, T4 DNA polymerase and *S. solfataricus* Pol B1 all have a slightly longer Exo-loop with a conserved motif, (F/I)P(D/E), located in the center of the loop. Interestingly, P301 is in a structurally conserved position among these very diverse family B polymerases with a low sequence identity. (2) In other archaeal polymerases, *P. abyssi* DNA polymerase, *P. furiousus* DNA polymerase and Deep Vent DNA polymerase (from *Pyrococcus* strain GB-D), the Exo-loop is slightly shorter, having instead a conserved EEF-motif and taking a different conformation than the eukaryotic family B polymerases (Fig. 3a). The three reported editing mode structures, one archaeal^37^ and two bacteriophage family B polymerases^22,38^, suggested that the orientation of the terminal base in the insertion pocket varies between structures, and that the structure of the Exo-loop might affect the orientation of the terminal base in the pocket. Based on the structural comparisons, we chose to fit the three terminal nucleotides of ssDNA from the structure of RB69 gp43 in editing mode (PDB 1clq [https://www.rcsb.org/structure/1clq]^22^) into our Pol2 wild-type exonuclease domain structure, and used this as a starting point for the simulations.

### Simulations suggest R301 interferes with binding of ssDNA

Molecular dynamics simulations were performed on the X-ray structures of the wild-type and P301R variant of the exonuclease domain (residues 286–488) in complex with a 3-nt long ssDNA. The wild-type and cancer variant were compared with respect to: (1) how the ssDNA would fit into the exonuclease site, (2) what conformations are accessible to the terminal nucleotide at the 3′-end of the ssDNA, and (3) how the ssDNA affects the conformation of the Exo-loop.

**Fig. 4:**
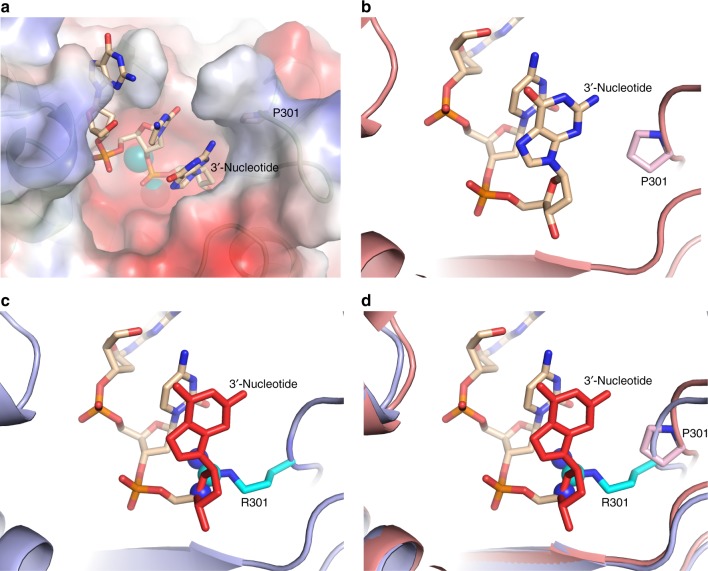
Modelling DNA into the exonuclease site of Pol2_CORE_. **a** The electrostatic surface view of the wild-type exonuclease domain of Pol2_CORE_ with the modeled position of single stranded DNA (ssDNA) in the exonuclease active site. A representative structure of the ssDNA from our molecular dynamics simulations is shown as sticks (wheat color). **b** A representative structure of DNA in the wild-type exonuclease domain of Pol2_CORE_ from the molecular dynamics simulation in cartoon view. The 3´-end of the ssDNA is shown in the front with P301 (in light pink) in the exo-loop on the right hand side. **c** The crystal structure of P301R overlayed with the ssDNA from (**b**). The terminal nucleotide (in red) occupies the same space as R301 (the atoms in the guanidine group shown as spheres). **d** The crystal structure of P301R overlayed with the best representative model from molecular dynamics simulations with wild-type exonuclease domain and ssDNA from (**b**). The terminal nucleotide (in red) occupies the same space as R301 (with the guanidine group shown as spheres)

**Fig. 5:**
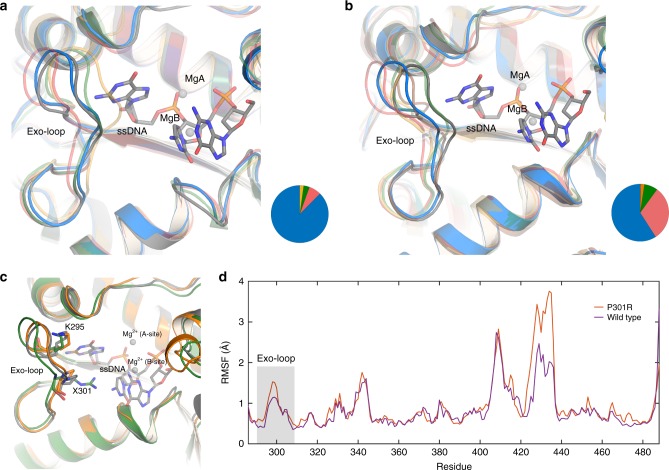
A comparison of different representative clusters of the Exo-loop during 3 × 200 ns simulations of wild-type and P301R forms of the exonuclease domain of Pol ε. The structures of (**a**) wild-type and (**b**) P301R were obtained by performing average-linkage clustering with a sieve value of 5 and a cutoff distance of 2.0 Å. Shown here are the centroids from each cluster, illustrating the greater conformational variability of the Exo-loop in the P301R mutant. The pie charts denote the relative population of each cluster, and have been color matched to the different structures shown in the overlay panels (**a**, **b**). The actual corresponding cluster occupancies are shown in Supplementary Table 1. The starting structure based on the crystal structure and ssDNA and metals from RB69 gp43 (PDB ID: 1clq [https://www.rcsb.org/structure/1clq]) used for simulations is depicted in gray color. **c** An overlay of the centroid of the most populated cluster for each set of simulations shows that the Exo-loop is pushed out of the active site in the P301R variant (green) compared to the wild-type enzyme (orange). The starting structure based on the crystal structure and ssDNA and metals from RB69 gp43 (PDB ID: 1clq [https://www.rcsb.org/structure/1clq]) used for simulations is depicted in gray. **d** A comparison of the root mean square fluctuations (RMSF) of all C_α_-atoms in our system during each set of simulations also highlights the slightly larger conformational flexibility of the Exo-loop in the P301R variant

**Fig. 6:**
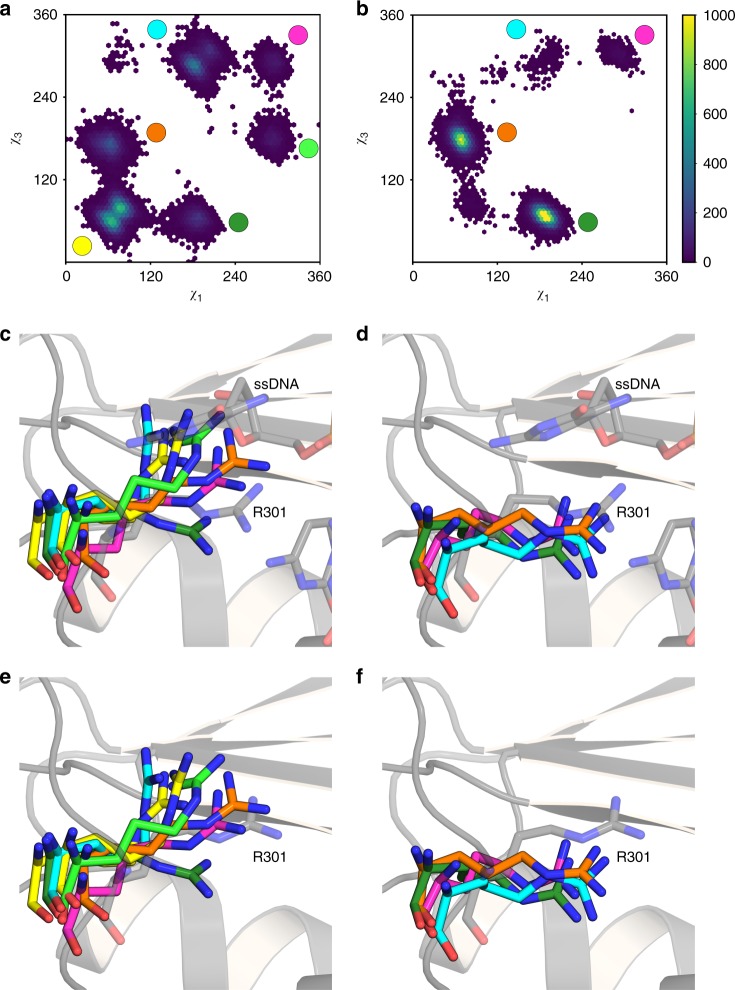
Population distributions of the χ_1_ and χ_3_ dihedral angles (°) of the R301 side chain of the P301R mutant forms of the exonuclease domain of Pol ε. Τhe dihedral angles formed by N–C_α_–C_β_–C_γ_ and C_β_–C_γ_–C_δ_–N_ε_, respectively. The colors shown in the heat maps correspond to frequencies each value is sampled during the simulation. Shown here are the population distributions from (**a**) the apo-enzyme and (**b**) the enzyme in complex with ssDNA. As can be seen, the R301 side chain takes multiple conformations during each simulation. Selected conformations of this sidechain, corresponding to structures from highly populated clusters in (**a**, **b**) are shown in (**c**, **d**) for the apo-enzyme and the enzyme in complex with ssDNA, respectively, with the corresponding regions in (**a**, **b**) marked by circles in the same color as the side-chains. The starting structure used for simulations is depicted in gray color. Note that in the case of the structures from the simulations of the apo-enzyme (**c**), we have placed the ssDNA in the background figure in gray as a point of reference only. The selected conformations of the R301 side chain in (**e**, **f**) are identical to (**c**, **d**) but with the background figure replaced by the crystal structure of P301R in gray as a point of reference

The limited structural differences between the wild-type enzyme and the P301R variant allowed us to superimpose the wild-type exo-domain-ssDNA model (Fig. 4a, b) with the P301R structure (Fig. 2b) to examine if the ssDNA could fit in the P301R structure. Interestingly, we observe a steric clash between the terminal residue of the ssDNA and R301 (Fig. 4c, d), suggesting that R301 will interfere with the binding of ssDNA to the exonuclease site. This steric hindrance likely explains the severe reduction of exonuclease activity observed with the catalytic fragment of human Pol ε-P286R^17^ and the four-subunit yeast Pol ε-P301R (see companion paper by Xing et al.^39^.). We confirmed that yeast Pol2_CORE_-P301R used in this work has a similar strong exonuclease defect (Supplementary Figure 2). In all three studies, however, the cancer variant showed a weak residual exonuclease activity. This led us to ask if R301 might be conformationally variable. Therefore, as our starting point, we studied the flexibility of the R301 side chain both in the absence (apo-form) and presence of the ssDNA, from our simulation data. To facilitate the latter, we moved R301 to a starting position where the ssDNA would not clash with R301 before the simulation started, in order to be able to place the ssDNA into the active site. This was achieved by performing geometry minimization in Maestro^40^ to remove the steric clash, in order to see whether the simulation would accept that ssDNA was bound in the active site if the steric clash between the ssDNA and the R301 side chain is removed. In these simulations, the exo-loop was slightly pushed out compared to the crystal structure (Fig. 5). However, R301 was maintained in a conformation that allows ssDNA binding to the catalytic site during three independent 200 ns simulations (600 ns total simulation time) (Fig. 5b). To verify that this is not simply an artefact of having manually removed the steric clash between the R301 side chain and the ssDNA, we performed a population distribution analysis of the χ_1_ and χ_3_ dihedral angles of the R301 side chain (corresponding to the dihedrals formed by N–C_α_–C_β_–C_γ_ and C_β_–C_γ_–C_δ_–N_ε_, respectively) during simulations of both the apo-form of the P301R variant, as well as the P301R variant in complex with the ssDNA. This analysis showed that the R301 side chain can take multiple distinct conformations in both the apo-form of the enzyme as well as in the complex with the ssDNA (Fig. 6). The most populated conformations sampled by the R301 side chain during our simulations of the apo-form correspond to conformations that would create a steric clash with the ssDNA (Fig. 6a–c). However, even in the simulations of the apo-form of the enzyme, the R301 side chain occasionally does sample conformations that would allow for the binding of the ssDNA, including the rotamer used in the simulations in this work (see green rotamer in Fig. 6c). These data indicate that R301 is flexible, and can adopt various conformations that could permit binding of ssDNA at the exonuclease site, even if this is likely to be a rare event.

**Fig. 7:**
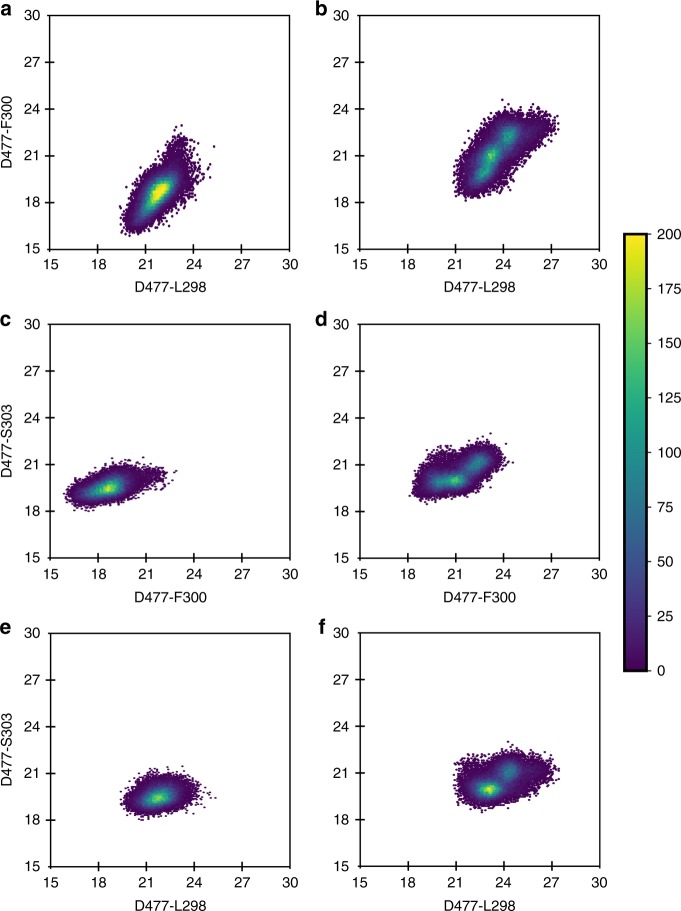
Conformational space sampled by the Exo-loop in the wild-type and P301R mutant forms of the exonuclease domain of Pol ε. The data were obtained based on analysis of the population distributions of the distances (Å) between the C_α_-atoms of residues L298, F300, S303 to the C_α_-atom D477. These residues were chosen in order to be able to compare the position of specific residues on the flexible Exo-loop to a relatively rigid point on the protein, providing information both on the conformational space sampled by the loop and also whether the shape of the loop changes during the simulations. The results are shown for the wild-type in (**a**, **c**, **e**), and for the P301R mutant in (**b**, **d**, **f**). The colors shown in the heat maps correspond to the frequencies with which each value is sampled during the simulation

We next examined the conformational flexibility of the Exo-loop in the different simulations. To facilitate this, we monitored the sampling of the distances between the C_α_-atoms of L298, F300 and S303 and the C_α_-atom of D477 in simulations of both the wild-type enzyme and P301R variant (Fig. 7). This allowed us to compare the movement of the flexible parts of the Exo-loop with respect to a relatively fixed part of the enzyme during our simulations. Three observations were made from this analysis: (1) The conformational space sampled by the Exo-loop is not vastly different between the wild-type and mutant enzymes. Therefore, the overall conformation of the loop does not change during the simulations. (2) The distances sampled during simulations of the P301R variant are around 3–4 Å greater than those sampled in the simulations of the wild-type enzyme. This shows that the loop is displaced and more open than its position in the wild-type crystal structure. Finally, (3) there is a single conformational cluster for each simulation, which is more well-defined for the wild-type enzyme, whereas in the case of the P301R variant, it is more widely distributed over conformational space. This argues that the Exo-loop is more flexible in the mutant enzyme, as also observed in the plots of the root mean square fluctuations (RMSF) of the backbone C_α_-atoms during our simulations (Fig. 5d). We note here that the much larger peak seen at residues 420–440 in Fig. 5d is due to the fact that these residues are part of a loop at the interface between the exonuclease domain and the rest of the protein in the complete macromolecule of DNA Pol ε. However, as our molecular dynamics simulations were performed on only the exonuclease domain, these stabilizing interactions are absent, allowing the loop to exhibit greater flexibility. While this is also interesting, we believe that the change in RMSF for the Exo-loop is more functionally relevant, as it is located close to the active site, thereby directly impacting its shape and size. Specifically, the representative conformations shown in Fig. 5a, b illustrate that, in the mutant enzyme, the Exo-loop moves away from its wild-type conformation to accommodate the long arginine side chain.

**Fig. 8:**
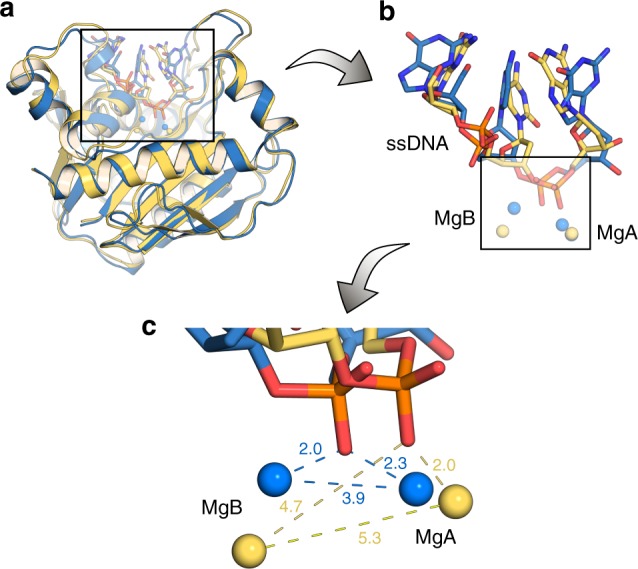
Comparisons of the exonuclease active site of wild-type and P301R Pol2_CORE_ after molecular dynamics simulations. Overlay of structures of the exonuclease active site of wild-type (blue) and P301R Pol2_CORE_ (yellow), taken as representative snapshots from 3 × 200 ns simulations of each system, and zooming in progressively from (**a**) the full tertiary structure to (**b**) the exonuclease-site to (**c**) the metal binding site. As can be seen, the proposed bridging conformation of the phosphate group from the ssDNA backbone is lost in the P301R variant, due to displacement of the Mg^2+^ ion in the B-site. The distances annotated in (**c**) are average distances (Å) over the entire simulation and all replicas

As a last point, we note that in the catalytic site itself, the 3′ terminal base was more flexible in the presence of R301, deviating significantly from its position in the starting structure. Clustering analysis on the ssDNA (see the Methods section) showed that there were 2–3 distinct conformations of the ssDNA that were more prominent than the others in the P301R variant. In contrast, only one conformation of the ssDNA was predominant in the wild-type enzyme (Supplementary Table 1). This shows that the nitrogenous base of the terminal nucleotide can potentially reorganize itself to avoid steric clashes with the bulky R301 sidechain. However, such a reorganization could lead to subtle conformational changes in the backbone leading to an observed loss of the bridging of the phosphate with both the catalytic metal ions (Fig. 8). That is, while Mg^2+^ ions at both the A and B sites were found to be bridged by the backbone phosphate oxygen of the DNA in the wild-type enzyme, only the ion at the A-site coordinated with the phosphate oxygen in P301R. Both metal ions, and in particular the B-site ion, were displaced compared to their positions in the wild-type structure, leading to a larger metal-metal distance as well as the loss of the bridging conformation of the backbone phosphate oxygen. This is in sharp contrast to simulations of the wild-type exonuclease domain where this bridging conformation is maintained throughout the simulation.

Taken together, the structural and molecular dynamic simulation data show that the mechanism of exonuclease inactivation in Pol2_CORE_-P301R involves a steric obstruction of ssDNA binding in the exonuclease site, in addition to the loss of catalytic activity. As discussed below, we propose that the steric obstruction is the key to understanding the differences between Pol ε-P301R and the catalytic-inactive Pol ε-D290A,E292A enzymes and the extraordinary pathogenic effects of the cancer-associated variant.

## Discussion

*POLE* mutant tumors are widely labeled as proofreading-deficient, but the true mechanistic basis for the pathogenicity of *POLE* variants has been uncertain. Mutations in tumors preferentially affect non-catalytic residues in the exonuclease domain, with P286R being the most common variant^12,13^. In the yeast model, the P286R analog causes a nearly two orders of magnitude stronger increase in mutagenesis than the exonuclease-inactive Pol ε variant lacking the catalytic residues D290 and E292^18^. Mice carrying a germline *Pole*^*P286R*^ allele also have much stronger mutator and cancer-prone phenotypes than exonuclease-deficient *Pole* mice lacking the two catalytic residues^19^. Thus, all available evidence indicates that there is a major distinction between the effects of the cancer variant and a simple inactivation of Pol ε exonuclease activity. We describe the crystal structure of the yeast Pol ε-P286R analog and the molecular dynamic simulation analysis that provide insight into the nature of this distinction, which has previously been elusive.

The crystal structure and molecular dynamics simulations suggest that R301 is at large positioned in a conformation that would interfere with the binding of the 3´-end of the nascent strand to the exonuclease active site. We do note that R301 is conformationally dynamic, and, on rare occasions, might adopt a conformation that allows the ssDNA to enter the catalytic site (Fig. 6). However, even when simulating such a rare-event scenario, we observe that the incorporation of the ssDNA severely disturbs the catalytic site, leading to a significant displacement of the metal ion at the B-site and loss of the preferred catalytic orientation of the phosphate, thus leading to the impaired catalytic activity of this variant even when ssDNA is bound. Taken together, therefore, our analysis indicates that the ssDNA is unlikely to bind to the exonuclease active site due to the presence of R301, and even if the 3´-end was bound, the catalytic efficiency would be very low. These findings are consistent with the biochemical data showing severely impaired exonuclease activity of human Pol ε-P286R and yeast Pol ε-P301R (^17,39^, and Supplementary Fig. 2). Importantly, this mechanism of inhibition of the exonuclease activity differs from the catalytically inactive Pol ε (D290A,E292A) that will accept ssDNA in the exonuclease site but cannot perform hydrolysis as the catalytic ions are not associated with the exonuclease active site. The impact of the catalytic ions on the binding of ssDNA to the active site will be minor, as it was earlier shown that the capacity of the RB69 gp43 wild-type exonuclease domain to bind a mismatched primer only decreased by 25% when two catalytic amino acids were exchanged for alanine^40^.

The distinct mechanism of exonuclease inactivation by the P301R and the catalytic residue mutations has implications for understanding the higher pathogenicity of the cancer variant. The fidelity of DNA synthesis by DNA polymerases with proofreading activity is determined by a delicate balance between the forward polymerization and the reverse proofreading reaction. Altering this balance will decrease or increase the propensity of the DNA polymerase to extend DNA synthesis from mis-incorporated nucleotides or bypass DNA lesions in an error-prone manner. This is illustrated by detailed kinetic studies of the T4 DNA polymerase, a model family B polymerase. The kinetic scheme for the exonuclease pathway of T4 DNA polymerase involves at least four steps with different kinetic rates^41^. The insertion of a correct nucleotide is done at a very fast rate. This rate varies between replicative DNA polymerases, but is for T4 DNA polymerase and Pol ε in the range of 300 s^−1^ or faster^42,43^. The rate is, however, significantly slowed down if the DNA polymerase attempts to extend from an incorrectly inserted nucleotide or if there is a DNA lesion in the template strand (Step 1). The slowed down DNA polymerase reaction allows sufficient time to overcome the kinetic barrier that normally does not allow the 3´-end of the nascent strand to be transferred to the exonuclease site, 30–40 Å away from the polymerase site. The fraying of the double-stranded DNA, the conformational change of domains in the polymerase, and binding to the exonuclease site occur at a rate that is slower than the extension from a correct nucleotide but faster than the extension from a mismatch (Step 2). The bound ssDNA in the exonuclease site is hydrolyzed at a very fast rate, e.g. 100 s^−1^ for T4 DNA polymerase and 65 s^−1^ for Pol ε^42,43^ (Step 3). The last step is the fastest step when the hydrolyzed 3´-end is transferred back to the polymerase site (Step 4). Estimates for T4 DNA polymerase suggest that the binding of ssDNA to the exonuclease site is the rate-limiting step regardless of whether there is a direct transfer to the exonuclease site or if the DNA polymerase first dissociates before binding to the exonuclease-site^44^. In accordance with the essential role of the exonuclease reaction in the fidelity of DNA synthesis, DNA polymerase alterations that shift the balance between exonuclease and polymerase activity toward the forward polymerase reaction produce mutator enzymes^41^. In the case of Pol ε-P301R, the inability to properly bind DNA in the exonuclease site is expected to increase the kinetic barrier to form a ssDNA-exonuclease domain complex. As a consequence, the difference in rate between proofreading and extension decreases, enhancing the probability for P301R to extend mismatched primer termini and ultimately resulting in a high rate of base substitutions. In contrast, the elimination of catalytic residues in Pol ε-D290A,E292A is not expected to affect the formation of a ssDNA-exonuclease domain complex, which, in the presence of a mismatched primer terminus, would keep the extension rate low and restrict mutagenesis. In agreement with this model, the companion study by Xing et al.^39^ demonstrates greatly increased mismatch extension ability of Pol ε-P301R in comparison to the exonuclease-inactive Pol ε-D290A,E292A. In addition to mismatch extension, the shift toward DNA synthesis in Pol ε-P301R could potentially also increase DNA lesion bypass by Pol ε. We have shown previously that wild-type Pol ε has an unexpectedly high capacity to bypass abasic sites in the DNA template under single-hit conditions^45^. It is possible that P301R substitution further enhances this capacity and the mutagenic consequences of DNA damage, which would add to any mutator effect from error-prone copying of undamaged DNA.

Considering that Pol δ is responsible for the bulk synthesis of DNA on the lagging strand and Pol ε on the leading strand, it would seem likely that the corresponding proline in the exo-loop of Pol δ would also be frequently altered in human tumors. While there may be several explanations as to why this is not the case, one plausible explanation is related to the distinct biochemical properties of Pol ε and Pol δ. Pol ε has a unique domain, the P-domain, that encircles the newly synthesized double-stranded DNA^26^ and gives Pol ε much higher intrinsic processivity compared to Pol δ^46^. Thus, it is less likely that Pol ε will dissociate from the template while proofreading the DNA, when compared to Pol δ and other family B polymerases. The increased mismatch extension ability of Polε-P301R could conceivably synergize with the high processivity to result in an exceptionally strong increase in the error rate. This synergistic increase would not be observed in Pol δ because its processivity is low. Even though Pol δ is tethered to the template by the clamp (PCNA), it should be noted that Pol δ is designed to transiently dissociate from the template to perform its function in Okazaki fragment maturation^47^. Indeed, in vivo studies in the yeast system have shown that errors made by exonuclease-deficient Pol δ are easily accessible to correction by wild-type Pol δ molecules in heterozygous diploid strains, and, as a consequence, Pol δ exonuclease defects are almost completely recessive^48,49^.

To summarize, our structural and molecular dynamics simulation studies revealed that the cancer-associated P301R substitution prevents proper positioning of ssDNA in the exonuclease active site of Pol ε. This steric hindrance appears to be the key structural alteration that distinguishes the cancer variant from the catalytic residue variant Pol ε-D290A,E292A, which completely lacks the proofreading activity but is not nearly as mutagenic as Pol ε-P301R. We propose that the inability to bind DNA at the exonuclease site constitutes the mechanistic basis for the extremely high mutator effect of Pol ε-P301R and its mammalian counterpart. The lack of partitioning of the DNA between the polymerase and exonuclease sites is expected to strongly promote the extension of mismatched primer termini, as, indeed, observed with the four-subunit Pol ε-P301R in the companion study^39^. Future studies could address whether the defective complex formation with ssDNA in the exonuclease site also increases the contribution of Pol ε to mutagenic bypass of DNA lesions, and whether the extraordinary mutator effects of the proline-to-arginine substitution will be unique to Pol ε due to the high intrinsic processivity of this polymerase.

## Methods

### Over-expression and purification of proteins

The mutant clones were constructed by site-directed mutagenesis in the pET28a vector containing the catalytic part of the Pol2 subunit of Pol ε (residues 1–1187) (a kind gift from Aneel K. Aggarwal^50^). We used the *E. coli* expression system to express and purify the protein. We purified 6xHis-tagged protein by binding to Ni^2+^-NTA beads in 25 mM Tris-Ac pH 7.5, 10*%* Glycerol and 300 mM NaAc, called buffer B_300_ (300 denotes NaAc concentration), and the eluted fractions were incubated with PreScission protease to remove the His-tag. The protein sample was then passed through Ni^2+^-NTA beads a second time to isolate the protein without the tag. The eluted protein was purified further on a 1 ml Mono-Q column with a linear gradient (B_200_–B_1000_). The peak fractions were pooled and the buffer was adjusted to B_800_ over a PD10 desalting column from Sigma-Aldrich.

### Crystallization and data collection

The ternary complex of the catalytic part of the Pol2 subunit was formed with 11ddC/16 primer–template (Supplementary Table 2) with dT as the templating base, and dATP as the incoming nucleotide^26^. The protein–DNA complex (1:1.2) was prepared in the presence of Ca^2+^ to inhibit DNA degradation by the exonuclease domain. Crystals were obtained under new crystallization conditions containing 50 mM MES, 150 mM NaAc and 8*%* PEG20K in the reservoir. Hanging-drop vapor diffusion technique was used to obtain the crystals. For data collection at −80 °C, the crystal was frozen in liquid nitrogen after it was equilibrated with the well solution containing 15*%* glycerol. The data sets for Pol2_CORE_-P301R and Pol2_CORE_-M644G were collected at 100 K on beamline ID23 at ESRF (Grenoble, France). Since crystals were obtained under new conditions, the unit cell dimensions were different from previous Pol2_CORE_ crystal structures 4m8o[https://www.rcsb.org/structure/4m8o]^26^ and 4ptf[https://www.rcsb.org/structure/4ptf]^27^. Two P301R mutant crystals diffracted to 2.62 Å and 2.65 Å with space group C2 and P2, respectively (Table 1). The crystal for Pol2_CORE_ M644G containing the wild-type exonuclease domain diffracted to 2.5 Å with space group P2 (Table 1).

### Structure determination and refinement

Phaser^51^ was used to solve the structure of Pol2_CORE_-P301R by molecular replacement technique using 4m8o[https://www.rcsb.org/structure/4m8o]^26^ as the molecular replacement model with a single ternary complex in the asymmetric unit. The Pol2_CORE_-M644G dataset processed in P2 space group gave a Mathews coefficient (V_M_) of 2.78Å^2^ Da^−1^ with 56% solvent content, which suggested two ternary complexes in the asymmetric unit. Two macromolecules of ternary complex of Pol2_CORE_ were searched for using 4m8o[https://www.rcsb.org/structure/4m8o]^26^ as the molecular replacement model. Coot^52^ and the Phenix package^53^ were used for model building and refinement of the structures, respectively. In the refined structures of Pol2_CORE_-P301R (both 6g0a[https://www.rcsb.org/structure/6g0a] and 6i8a[https://www.rcsb.org/structure/6i8a]) and Pol2_CORE_-M644G (6fwk[https://www.rcsb.org/structure/6fwk]), 99.7*%* and 99.8*%* of the residues, respectively, were in favored or allowed regions of the Ramachandran plot. The models were validated by using Coot^52^ and MolProbity^54^. PyMOL was used to superimpose the structures. Structure-based sequence alignment was created using UCSF Chimera^55^. The crystal structure of Pol2_CORE_ M644G containing the wild-type exonuclease domain at 2.5 Å was superimposed with 4m8o[https://www.rcsb.org/structure/4m8o]^26^ and 4ptf[https://www.rcsb.org/structure/4ptf]^27^ with a root mean square deviation (r.m.s.d.) of 0.35 Å for 975 C_α_ atoms. The structures are thus not affected by the M644G change in the palm domain, which allowed us to use its wild-type exonuclease domain structure as a starting model for the molecular dynamics study (see the Results section). The Pol2_CORE_-P301R structure with PDB ID 6g0a[https://www.rcsb.org/structure/6g0a] is depicted in the figures.

### Exonuclease assay

The primer–templates were prepared by mixing 6 μM primer strand with 7.2 μM template strand in a buffer containing 100 mM Tris-HCl pH 7.5 and 100 mM NaCl, heated to 90 °C for 5 min in a heating block and, thereafter, slowly cooled to room temperature. The oligonucleotides are listed in Supplementary Table 2. Exonuclease assays were performed essentially as described in Hogg et al.^26^. Briefly, for each time-point a 10 μL reaction mix A (10 nM TET-50/80-mer, 20 mM Tris-HCl pH 7.8, 0.1 mg mL^−1^ BSA, 80 mM NaAc, 0.5 mM DTT and 5, 10 or 20 nM Pol ε) was pre-incubated on ice and then mixed with 10 μL reaction mix B (16 mM MgAc, 20 mM Tris-HCl pH 7.8, 0.1 mg mL^−1^ BSA, 0.5 mM DTT) to start the reaction that was incubated at 30 **°**C for the indicated times in Supplementary Figure 1. The reactions were terminated by the addition of 20 μL of 95% formamide, 20 mM EDTA, and 0.1% bromophenol blue, heated to 85 °C, and 5 μl of each reaction was loaded onto a 10% polyacrylamide gel containing 7 M urea and 25% formamide in 1x TBE. The gel was scanned with a Typhoon Scanner 9400 (GE Healthcare) at the Alexa 532 nm setting to excite the fluorophore, tetrachlorofluorescein, that was covalently bound to the 5′ end of the primer.

### Molecular dynamics simulations

Molecular dynamics simulations were performed on the wild-type and P301R variants of the exonuclease domain (residues 286–488) of DNA polymerase ε (PDB ID: 6fwk[https://www.rcsb.org/structure/6fwk] and PDB ID: 6g0a[https://www.rcsb.org/structure/6g0a]). The structures comprise the protein domain and two Ca^2+^ ions. For the simulations, the two Ca^2+^ ions were manually replaced by the physiologically relevant Mg^2+^ ions. The catalytic Mg^2+^ ions were replaced by octahedral dummy models, using the parameters described by Duarte and co-workers^56^, in order to capture both the electrostatic and structural properties of the metal ion without needing artificial restraints or bonds. The X-ray crystal structure of a replicative DNA polymerase editing complex (PDB ID: 1clq[https://www.rcsb.org/structure/1clq]^22^) was used as a reference to extract coordinates of the terminal DNA nucleotides, as well as of the metal ions, which exist in a slightly different position in space compared to those in the unliganded apo-structures. The coordinates were extracted by alignment of the coordinates of the replicative DNA polymerase on that of the wild-type exonuclease domain. The spatial coordinates of the metal ions and three terminal nucleotides from the 3′-end of the DNA at the active site of the DNA polymerase editing complex (PDB ID: 1clq [https://www.rcsb.org/structure/1clq]^22^) were used to manually place the 3′-GCG-5′ single stranded DNA (ssDNA) sequence in the active sites of the wild-type and P301R variants of the exonuclease domain. Bad steric contacts between the guanine base of 3′-G and the sidechain atoms of K295 in wild-type exonuclease domain, and between R301 and the ssDNA backbone atoms were removed using the geometry clean-up tool implemented in Maestro v. 11.1.012 (Release 2017–1)^57^. An apo form of P301R exonuclease domain was also generated by deleting the ssDNA from the starting structure used for the ssDNA-bound structure.

All MD simulations were performed using the AMBER16 simulation package^58^. The ff14SB force field^59,60^ was used for the protein atoms, whereas the Parmbsc1 force field^61^ as implemented in AMBER16 was used for the DNA atoms. The PMEMD module was used for the initial minimization, equilibration and production runs^62^. The topology and initial coordinates were generated with LEaP. The protein–DNA complex was placed in an octahedral solvent box comprised of TIP3P water molecules^63,64^, with the box extending at least 10 Å from the solute in each direction. Asp, Glu, Arg and Lys residues were kept in their ionized states while histidine residues were kept neutral protonated at the N_ε_-atom. The total system charge was neutralized by adding the appropriate number of Na^+^ counter ions to the system (15 for the wild-type enzyme, and 14 for the P301R variant). Additional Na^+^ and Cl^-^ ions were then added to the system to maintain an ionic strength of 0.15 M in the system.

The solvated system was subjected to a minimization procedure comprised of 2000 steps of steepest descent and 3000 steps of conjugate gradient minimization using 5 kcal mol^−1^ Å^−2^ harmonic positional restraints on all heavy atoms of the solute. This was followed by the following equilibration protocol. (1) 1.5 ns long NVT simulations were performed to increase the temperature of the system in three 500 ps steps: from 5 K to 100 K in the first, from 100 K to 200 K in the second, and from 200 to 300 K in the third step. (2) A 1 ns NPT equilibration was performed at a constant isotropic pressure of 1 atm in five steps of 200 ps length each, progressively decreasing the harmonic positional restraints on the solute heavy atoms from 5 kcal mol^−1^ Å^−2^ to 1 kcal mol^−1^ Å^−2^. Finally, (3) a 500 ps long NPT simulation was performed without any restraints on the system, resulting in an overall equilibration time of 3 ns. All simulations were performed using the Berendsen thermostat^65^ and pressure control algorithms, using a 1 ps time constant.

Three such independent equilibrations were performed on both the wild-type and P301R variants (with and without ssDNA) of the exonuclease domain system, each followed by a 200 ns long production simulation, thus resulting in three replicas for each variant. Each variant was sampled for a total simulation time of 600 ns per system (Supplementary Figure 3), giving rise to an overall simulation time of 1.8 µs over all systems. NPT conditions were used to perform all production simulations. Constant temperature (300 K) was maintained using the Langevin thermostat^66^ with a collision frequency of 2 ps^−1^. The Monte Carlo barostat^67^ was used to maintain a constant pressure of 1 atm. All bonds involving hydrogen atoms were constrained using the SHAKE algorithm^68,69^. A 10 Å cutoff radius was used to calculate short-range non-bonded interactions. Long-range electrostatic interactions were described using the particle mesh Ewald method^70,71^. All simulations were performed using a 1 fs time step, with snapshots saved every 2.5 ps.

### Analysis

Clustering analysis on the MD trajectories was performed using CPPTRAJ^72^. Simulation frames were extracted for every analysis every 12.5 ps of the trajectory, leading to the extraction of a total of 48,000 frames per system for the analysis. The Hierarchical-Agglomerative clustering algorithm was used with a sieve value of 5, and a cutoff distance of 2.0 Å. Measurements of dihedral angles, distances between atoms, and the calculations of root mean square deviations (RMSD), were performed using VMD 1.9.1^73^. GROMACS 2016.4^74^ was used to perform root mean square fluctuation (RMSF) calculations.

### Miscellaneous

Figures were prepared with PyMOL (http://www.pymol.org/).

### Code availability

All simulations in this work were performed using the AMBER16 simulation package. Further details about this simulation package, and how to obtain a license to the latest version of the code, can be found through the AMBER website: http://ambermd.org/

### Reporting Summary

Further information on experimental design is available in the Nature Research Reporting Summary linked to this article.

## Supplementary information

Supplementary Information

Reporting Summary

## Data Availability

A reporting summary for this Article is available as a Supplementary Information File. The data that support the findings of this study are available from the corresponding authors upon reasonable request. The atomic coordinates and structure factors have been deposit in the Protein Data Bank, www.rcsb.org[https://www.wwpdb.org/] with accession number 6fwk[https://www.rcsb.org/structure/6fwk], 6g0a[https://www.rcsb.org/structure/6g0a] and 6i8a[https://www.rcsb.org/structure/6i8a]). Input files, topology files, starting structures, and representative snapshots from the simulations are available from Dryad, 10.5061/dryad.1sb340g.
