# Supplementary material for: Structural consequence of the most frequently recurring cancer-associated substitution in DNA polymerase ε
Source: Nat Commun. 2019 Jan 22;10:373. doi: 10.1038/s41467-018-08114-9 (PMC6342957; doi:10.1038/s41467-018-08114-9)
Supplement: Supplementary file 1 — Supplementary Information [file 41467_2018_8114_MOESM1_ESM.pdf]

Supporting Information For:

Structural consequence of the most frequently recurring cancer associated substitution in DNA Polymerase epsilon.

Vimal Parkash[1], Yashraj Kulkarni[2], Josy ter Beek[1], Polina V. Shcherbakova[3], Shina Caroline Lynn Kamerlin[2*], and Erik Johansson[1]*

[1]Department of Medical Biochemistry and Biophysics, Umeå University, SE-90187 Umeå, Sweden

[2]Department of Chemistry - BMC, Uppsala University, Box 576, S-751 23 Uppsala, Sweden

[3]Eppley Institute for Research in Cancer and Allied Diseases, Fred & Pamela Buffett Cancer Center, University of Nebraska Medical Center, Omaha, NE 68198, USA

*Corresponding authors: erik.tm.johansson@umu.se, lynn.kamerlin@kemi.uu.se

Table of Contents

Supplementary Figures……………………..…………………………………………………S2-S4

Supplementary Figure S1; Overall structural comparison between Pol2$_{CORE}$ P301R and the previously published Pol2$_{CORE}$ structure..S2

Supplementary Figure S2: Exonuclease assays to compare the exonuclease activity between P301R and wild-type Pol2$_{CORE}$…………………………………………………………………S3

Supplementary Figure S3: Root mean square deviations (RMSD, Å) of all backbone C_{α}-atoms during molecular dynamics simulations of the exonuclease domain…………………......……S4

Supplementary Tables…………………….…………………………………………………S5-S6

Supplementary Table S1: Summary of cluster populations from simulations of the exonuclease domains of wild-type and P301R Pol2$_{CORE}$………...…………………………………………S5

Supplementary Table S2: Oligonucleotides used in the crystallization study………………..…S6

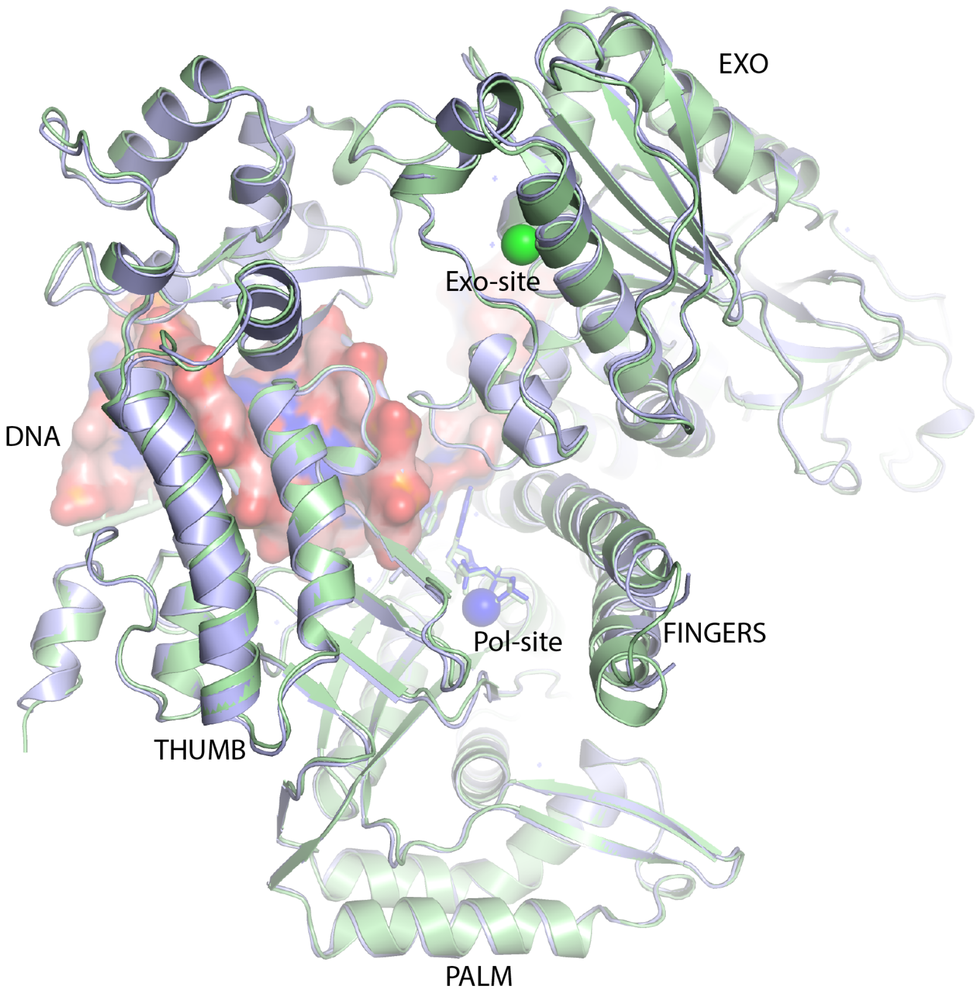

Supplementary Figure 1. **Overall structural comparison between Pol2$_{CORE}$ P301R (light blue) and the previously published Pol2$_{CORE}$ (pol2-4) structure (PDB ID: 4m8o) (pale green).** The domains of the polymerase are shown in cartoon representation, and DNA is in surface representation. The incoming nucleotide is in sticks, and the position of the polymerase active site is indicated as a blue sphere. The position of the exonuclease active site is indicated as a green sphere.

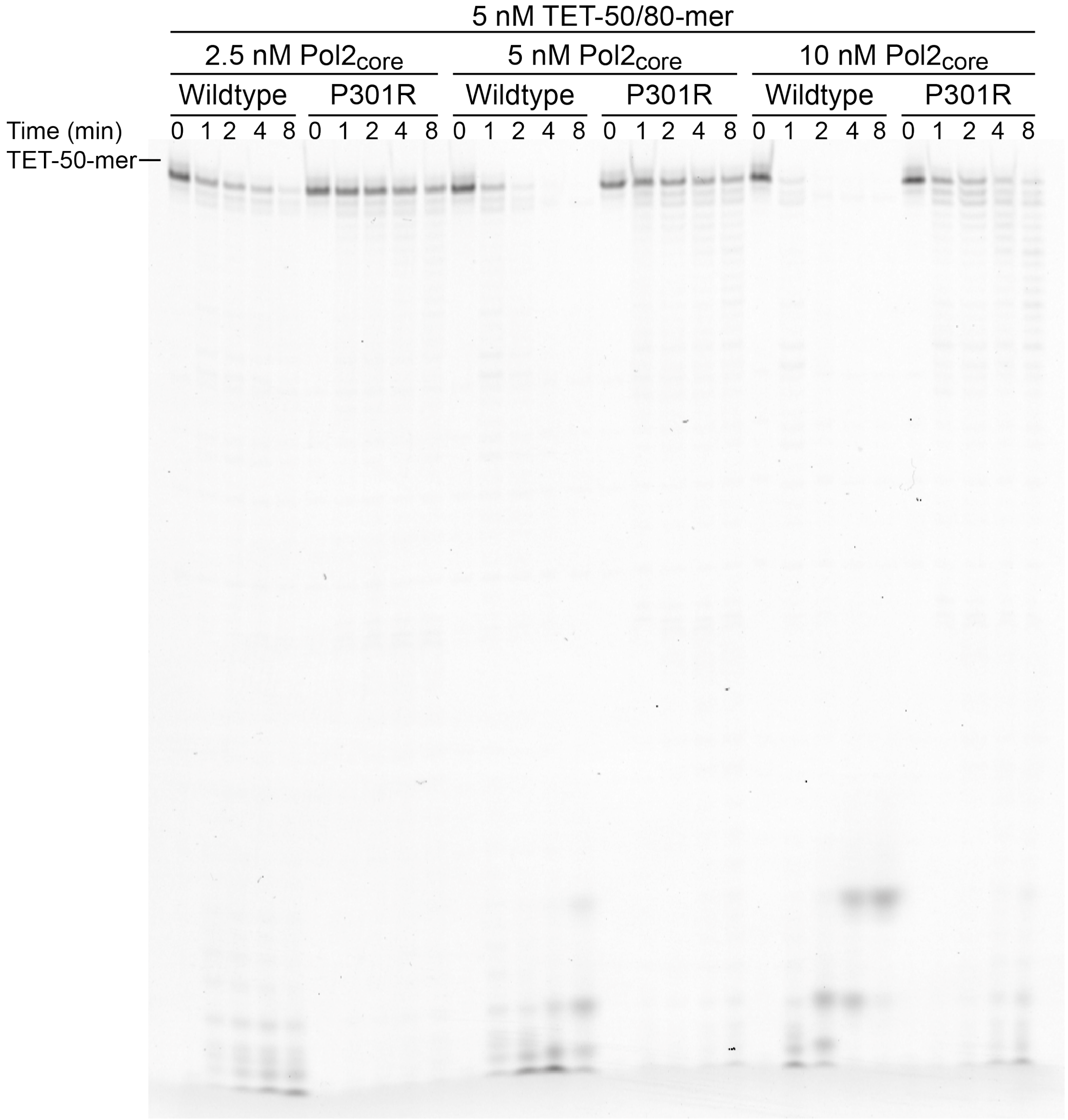

Supplementary Figure 2. **Exonuclease assays to compare the exonuclease activity of P301R and wild-type Pol2$_{core}$.** 5 nM DNA substrate was incubated with 2.5, 5 or 10 nM Pol2$_{core}$ over the indicated times at 30°C. Wild-type Pol2$_{core}$ rapidly degrades the primer into small fragments, seen at the bottom of the gel. In contrast, the P301R variant degrades a smaller fraction of the primer at all tested concentrations and most degradation products were longer than seen for the wild-type enzyme.

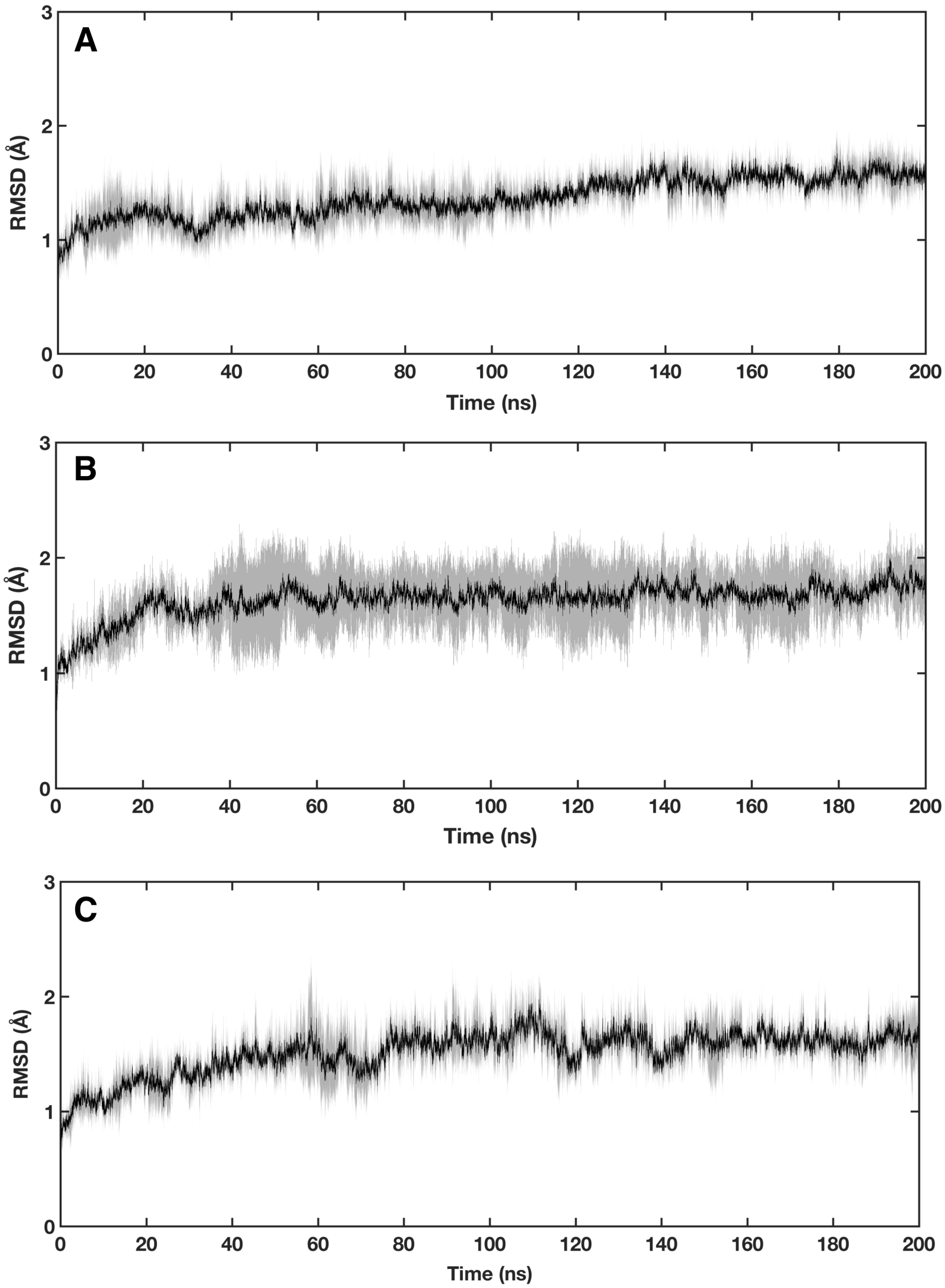

Supplementary Figure 3. **Root mean square deviations (RMSD, Å) of all backbone C_{α}-atoms during simulations of the exonuclease domain.** The data is shown for (A) wild-type and (B,C) P301R Pol2$_{CORE}$, in the (A,B) presence and (C) absence of ssDNA. All values are averages (solid lines) and standard deviations (shaded area) over 3x200 ns simulations per system, as described in the main text.

Supplementary Table 1. Summary of cluster populations from simulations of the exonuclease domains of wild-type and P301R Pol2$_{CORE}$.[a]

Variant	Cluster Rank	Frames	Occupancy	Cluster Rank	Frames	Occupancy
	Wild-Type			P301R		
**Protein**	1	38520	80.2 %	1	21571	44.9 %
	2	6950	14.5 %	2	13215	27.5 %
	3	2530	5.3 %	3	5780	12.0 %
				4	4945	10.3 %
				5	2489	5.2 %
**Exo-Loop**	1	42053	87.6 %	1	28415	59.2 %
	2	3292	6.9 %	2	14810	30.9 %
	3	1584	3.3 %	3	3652	7.6 %
	4	1071	2.2 %	4	995	2.1 %
				5	128	0.3 %
**ssDNA**	1	41098	85.6 %	1	20590	42.9 %
	2	6669	13.9 %	2	13332	27.8 %
	3	233	0.5 %	3	11694	24.4 %
				4	1067	2.2 %
				5	586	1.2 %
				6	502	1.0 %
				7	229	0.5 %

[a] For details of the clustering algorithm and parameters used to obtain this data, see the main text.

Supplementary Table 2: Oligonucleotides used in the crystallization study.

Primer KP11ddC	5’ TAACCGCGTTddC 3’
Template KT16	3’ ATTGGCGCAAGTTCTC 5’

Oligonucleotides used in exonuclease assay

TET-50-mer	5' GATCAGACTGTCCTTAGAGGATACTCGCTCGCAGCCGTCCA CTCAACTCA 3’
80-mer template	3' CTAGTCTGACAGGAATCTCCTATGAGCGAGCGTCGGCAGGT GAGTTGAGTAGGTCTTGTTGCAGTGACTGATAGTTCGAC 5'
