# Supplementary material for: Structural consequence of the most frequently recurring cancer-associated substitution in DNA polymerase ε
Source: Nat Commun. 2019 Jan 22;10:373. doi: 10.1038/s41467-018-08114-9 (PMC6342957; doi:10.1038/s41467-018-08114-9)
Supplement: Supplementary file 2 — Reporting Summary [file 41467_2018_8114_MOESM2_ESM.pdf]

nature research

Corresponding author(s): Shina C. L. Kamerlin and Erik Johansson

Reporting Summary

Nature Research wishes to improve the reproducibility of the work that we publish. This form provides structure for consistency and transparency in reporting. For further information on Nature Research policies, see Authors & Referees and the Editorial Policy Checklist.

Statistical parameters

When statistical analyses are reported, confirm that the following items are present in the relevant location (e.g. figure legend, table legend, main text, or Methods section).

n/a	Confirmed	
☒	☐	The exact sample size (*n*) for each experimental group/condition, given as a discrete number and unit of measurement
☒	☐	An indication of whether measurements were taken from distinct samples or whether the same sample was measured repeatedly
☒	☐	The statistical test(s) used AND whether they are one- or two-sided<br>*Only common tests should be described solely by name; describe more complex techniques in the Methods section.*
☒	☐	A description of all covariates tested
☒	☐	A description of any assumptions or corrections, such as tests of normality and adjustment for multiple comparisons
☒	☐	A full description of the statistics including central tendency (e.g. means) or other basic estimates (e.g. regression coefficient) AND variation (e.g. standard deviation) or associated estimates of uncertainty (e.g. confidence intervals)
☒	☐	For null hypothesis testing, the test statistic (e.g. *F*, *t*, *r*) with confidence intervals, effect sizes, degrees of freedom and *P* value noted<br>*Give P values as exact values whenever suitable.*
☒	☐	For Bayesian analysis, information on the choice of priors and Markov chain Monte Carlo settings
☒	☐	For hierarchical and complex designs, identification of the appropriate level for tests and full reporting of outcomes
☒	☐	Estimates of effect sizes (e.g. Cohen's *d*, Pearson's *r*), indicating how they were calculated
☒	☐	Clearly defined error bars<br>*State explicitly what error bars represent (e.g. SD, SE, CI)*

Our web collection on statistics for biologists may be useful.

Software and code

Policy information about availability of computer code

Data collection	1. Maestro v. 11.1.012 (Release 2017-1): Visualization program used for structure preparation before setting up the molecular dynamics (MD) simulations [commercial]<br>2. AMBER16: Simulation package used to perform MD simulations [commercial]<br>3. X-ray data was collected on beamline ID23 from ESRF using mxCUBE [commercial].
Data analysis	1. AMBER16: An implementation of the CPPTRAJ analysis code in this package used for extracting simulation frames from the MD trajectories. Clustering analysis was also performed using this tool. [commercial]<br>2. Visual Molecular Dynamics (VMD) version 1.9.1: Visualization program used for calculating root mean square deviations (RMSDs), and measurement of distances between atoms, and dihedral angles for subsequent analyses [open source]<br>3. GROMACS 2016.4: Simulation package used to calculate root mean square fluctuations (RMSFs) of residues during the simulations [open source]<br>4. Data analysis was done using all standard crystallographic softwares mentioned in the methods section.

For manuscripts utilizing custom algorithms or software that are central to the research but not yet described in published literature, software must be made available to editors/reviewers upon request. We strongly encourage code deposition in a community repository (e.g. GitHub). See the Nature Research guidelines for submitting code & software for further information.

Data

Policy information about availability of data

All manuscripts must include a data availability statement. This statement should provide the following information, where applicable:

- Accession codes, unique identifiers, or web links for publicly available datasets
- A list of figures that have associated raw data
- A description of any restrictions on data availability

The data that support the findings of this study are available from the corresponding authors upon reasonable request. The atomic coordinates and structure factors have been deposited in the Protein Data Bank, www.rcsb.org (PDB IDs: 6fwk, 6g0a and 6i8a). Input files, topology files, starting structures, and representative snapshots from the simulations are available from Dryad, DOI: XXX.

Field-specific reporting

Please select the best fit for your research. If you are not sure, read the appropriate sections before making your selection.

☒ Life sciences ☐ Behavioural & social sciences ☐ Ecological, evolutionary & environmental sciences

For a reference copy of the document with all sections, see nature.com/authors/policies/ReportingSummary-flat.pdf

Life sciences study design

All studies must disclose on these points even when the disclosure is negative.

Sample size	MD simulations: 200 ns of sampling was performed in each individual simulation. Three such simulations were carried out on each of the three systems studied computationally in this study. This resulted in a total sampling time of 1800 ns.
Data exclusions	MD simulations: No simulation data were excluded from analyses.
Replication	MD simulations: Three individual simulations were performed for each system. RMSD values were calculated and plotted with standard deviations to test for convergence of the simulations.
Randomization	MD simulations: Three replicates of simulations, each provided with a different starting velocity, were performed for each system. This was done to enhance data sampling and prevent bias towards a particular trajectory.
Blinding	Blinding was not possible. The study did not involve any human research participants. Experiments in the laboratory were carried out on bacterial cultures. Part of the study consisted of computational modeling of the protein complex.

Reporting for specific materials, systems and methods

Materials & experimental systems

n/a	Involved in the study
☒	☐ Unique biological materials
☒	☐ Antibodies
☒	☐ Eukaryotic cell lines
☒	☐ Palaeontology
☒	☐ Animals and other organisms
☒	☐ Human research participants

Methods

n/a	Involved in the study
☒	☐ ChIP-seq
☒	☐ Flow cytometry
☒	☐ MRI-based neuroimaging
